# Mechanochemical waves in focal adhesions during cell migration

**DOI:** 10.1126/sciadv.adw6425

**Published:** 2025-10-03

**Authors:** Marc A. Fernández-Yagüe, Elijah N. Marquez, Chetan S. Poojari, Jianping Fu, Yingxiao Wang, Aránzazu del Campo, Andrés J. García

**Affiliations:** ^1^ Petit Institute for Bioengineering and Bioscience, Georgia Institute of Technology, Atlanta, GA, USA.; ^2^ Woodruff School of Mechanical Engineering, Georgia Institute of Technology, Atlanta, GA, USA.; ^3^ Department of Chemistry, Queen Mary University of London, London, UK.; ^4^ School of Chemical and Biomolecular Engineering, Georgia Institute of Technology, Atlanta, GA, USA.; ^5^ Theoretical Physics and Center for Biophysics, Saarland University, Saarbrücken, Germany.; ^6^ Department of Mechanical Engineering, Department of Biomedical Engineering, Department of Cell and Developmental Biology, University of Michigan, Ann Arbor, MI, USA.; ^7^ Alfred E. Mann Department of Biomedical Engineering, University of Southern California, Los Angeles, CA, USA.; ^8^ INM – Leibniz Institute for New Materials, Saarbrücken, Germany.; ^9^ Chemistry Department, Saarland University, Saarbrücken, Germany.

## Abstract

Focal adhesions (FAs) are dynamic structures central to cell migration, serving as mechanotransduction sites linking the extracellular matrix (ECM) to intracellular signaling pathways such as FA kinase (FAK). How FAK becomes activated in response to cell-ECM adhesive forces at single FAs to facilitate directional motion is poorly understood. Using micropillar-based force microscopy and FA-targeted FRET biosensors, we monitored real-time traction forces and FAK activity at individual FAs during assembly and disassembly. Our results demonstrate oscillatory temporal coupling of traction force and FAK activity in high-tension FAs before FA disassembly. Cross-correlation analyses revealed that force precedes FAK activation, guiding FA turnover. Atomistic molecular simulations unveiled a force-induced mechanism where traction forces disrupt autoinhibitory FERM-kinase interactions in FAK, enabling catalytic activity without structural unfolding. Our findings provide mechanistic insights into the spatiotemporal integration of mechanical forces and biochemical signaling in cell migration.

## Teaser

Traction force and FAK signaling exhibit oscillatory temporal coupling in adhesive structures.

## INTRODUCTION

Mechanical forces influence development, homeostasis, tissue repair, and disease progression, including cancer and fibrosis (*1*–*4*). Focal adhesions (FAs), dynamic nanoscale complexes that connect the extracellular matrix (ECM) to the cell cytoskeleton, act as principal sites of mechanotransduction (*5*, *6*). Structural studies (*7*–*9*), genetic manipulation (*10*, *11*), and real-time imaging of cells treated with contractility inhibitors (*12*, *13*) have established that mechanical forces regulate FA assembly and identified FA kinase (FAK) as a key signaling molecule (*14*–*16*). FAK is a central transducer of forces and ECM stiffness, regulating mechanosignaling pathways, including yes-associated protein, transcriptional co-activator with PDZ-binding motif (YAP/TAZ), Ras homolog family member A (RhoA), Rho-associated coiled-coil containing protein kinase (ROCK), and piezo type mechanosensitive ion channel component 1 (Piezo1) (*17*). Force generation at FAs increases FAK phosphorylation and kinase activity, but the underlying mechanisms are poorly understood (*13*, *18*, *19*).

In the cytosol, FAK exists in a closed and autoinhibited conformation where its N-terminal band 4.1/ezrin/radixin/moesin homology (FERM) domain binds to the central catalytic domain to block the active site and key regulatory phosphorylation sites (*20*). During integrin-mediated cell adhesion to the ECM, the C-terminal FA targeting (FAT) domain targets FAK to the plasma membrane–apposed integrin signaling layer in FAs (*21*). Recent cryo–electron microscopy (cryo-EM) and biochemical studies show that binding of the FERM domain to the plasma membrane through phosphatidylinositol 4,5-bisphosphate [PI(4,5)P_2_] results in structural changes that release FAK autoinhibition, exposing the active site and regulatory phosphorylation residues, and induce FAK oligomerization (*20*). These priming steps are necessary for FAK autophosphorylation at Y397 and activation of full catalytic activity. Lietha and colleagues (*7*) speculated that actomyosin-driven forces at FAs could stretch the FAK molecule via FERM attachment to the plasma membrane and FAT anchoring to the cytoskeleton to expose FAK Y397 for phosphorylation and stimulation of catalytic activity. Single-molecule stretching experiments show that forces applied through the FERM and FAT domains disrupt FERM-kinase autoinhibition and expose the kinase domain. Furthermore, using cells adhering to deformable micropillar arrays, we demonstrated that the magnitudes of traction force and FAK localization as well as traction force and FAK Y397 phosphorylation are linearly coupled at individual FAs on stiff, but not soft, substrates, and FAK Y397 phosphorylation increases linearly with external forces applied to FAs using magnetic beads (*13*). However, it is not known how the dynamics of traction force and FAK activity evolve over the lifetime of FAs.

Here, we integrated micropillar-based traction force microscopy and a Förster resonance energy transfer (FRET)–based biosensor for FAK activity to analyze the time-resolved relationship between traction force and FAK activity during the assembly and disassembly of FAs. We discovered a synchronized oscillatory pattern of traction force and FAK activity in FAs under high levels of tension before FA disassembly, and temporal cross-correlation revealed that traction force precedes FAK activity at these complexes. Atomistic molecular simulations, with and without external force application, revealed a tension-mediated mechanism for separation of the FERM and kinase domains without unfolding of the FAK functional domains. These experimental and computational results provide previously unidentified insights into the conversion of mechanical forces into biochemical signals at FAs.

## RESULTS

### FAK biosensor to monitor real-time FAK activity at FAs

**Fig. 1.**
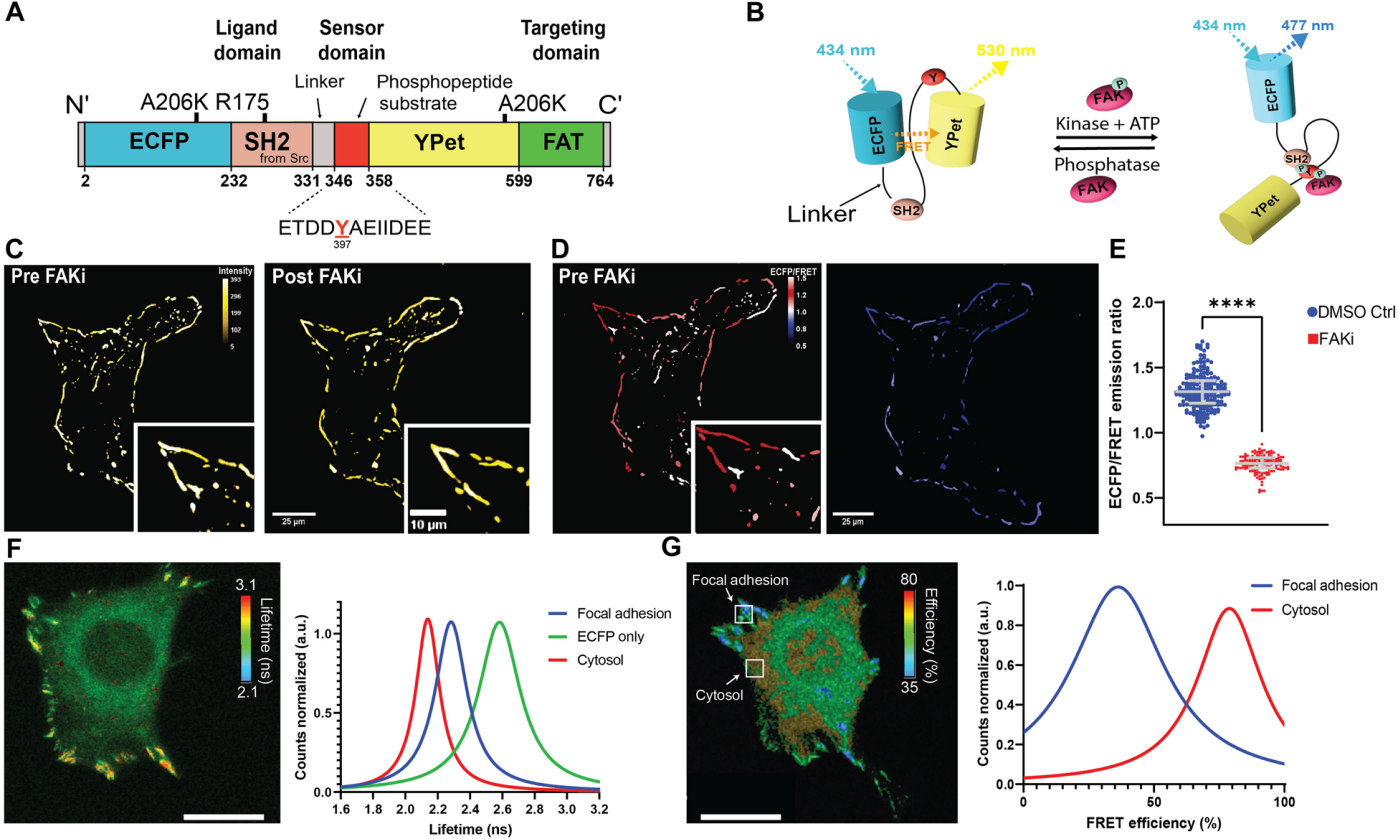
FRET biosensor for monitoring FAK activity at FAs. (**A**) The FAK biosensor is composed of ECFP, SH2 domain, flexible linker, FAK substrate peptide, YPet, and FAT domain. (**B**) Schematics illustrating the FRET effect of the FAK biosensor upon the actions of FAK phosphorylation or dephosphorylation. Upon phosphorylation of Y397 in the biosensor FAK substrate peptide, the SH2 domain forms an intramolecular complex with the phosphotyrosine side chain, increasing the distance between the FRET pair to alter the FRET signal. Dephosphorylation reverses the FRET change. ATP, adenosine 5′-triphosphate. (**C**) C-terminal FAT domain recruits the biosensor to FAs. YPet intensity showing slight changes before and after FAK inhibition (FAKi; 10 μM PF-573228, 60 min). (**D**) ECFP/FRET signal before and after FAKi (10 μM PF-573228, 60 min) showing that the biosensor is specific and sensitive to FAK activity. (**E**) EFCP/FRET signal at individual FAs [*n* = 195 FAs from seven cells across three independent experiments for FAKi (10 μM PF-573228, >60 min); *n* = 151 FAs from six cells across three independent experiments for DMSO control; means ± SD]. (**F**) Fluorescence lifetime image and quantification for fibroblasts expressing the FAK biosensor (*n* = 23 FAs from six cells across three independent experiments). Scale bar, 20 μm. (**G**) FRET efficiency image and quantification for FAs and cytosol (*n* = 19 FAs from five cells across three independent experiments). Scale bar, 20 μm. a.u., arbitrary units.

To monitor real-time FAK activity with high spatiotemporal resolution at individual FAs, we used a FRET biosensor with the FAK FAT domain (Fig. 1A) (*22*). The FAK biosensor contains the Src SH2 domain, a flexible linker (GSTSGSGKPGSGEGS) that allows for rotation between the two protein domains, and the substrate sequence containing Y397 of FAK (ETDD**Y**AEIIDEE). These structures are concatenated between the enhanced cyan fluorescent protein (ECFP) and yellow fluorescent protein (YPet) FRET pair, with YPet connected to the FAT domain for FA localization. Because ECFP and YPet can form antiparallel dimers, we introduced A206K mutations into ECFP and YPet to generate monomeric ECFP and YPet constructs and eliminate the unintended FRET resulting from intermolecular dimerization.

Prior studies validated the phosphorylation-induced intramolecular interaction between the SH2 domain and FAK substrate peptide as the only mechanism for the FRET response in this biosensor (*23*). When Y397 in the substrate of the biosensor is phosphorylated by active FAK (trans-activity), the substrate peptide can bind to the phosphopeptide-binding pocket of the SH2 domain and separate YPet from ECFP, thus decreasing the FRET signal and increasing the ECFP/FRET emission ratio (Fig. 1B). Therefore, the ECFP/FRET ratio can be used to monitor subcellular FAK activity. In vitro kinase assays revealed that no FRET changes occur when samples are exposed to active Src (*24*), confirming biosensor specificity toward FAK and not Src. In the present study, we used the FA-localized FAK biosensor to examine FAK activity at individual FAs during cell migration in mouse embryonic fibroblasts (MEFs). Using lentiviral constructs, we stably integrated the FAK biosensor construct into the MEF genome, enabling continuous monitoring and analysis of FAK activity with high sensitivity (Fig. 1C). Cells coexpressing the FAK biosensor and mCherry-paxillin demonstrated that the YPet signal of the FAK biosensor colocalizes with paxillin at FAs (fig. S1). The FAK biosensor preferentially localizes to FAs as reflected by the YPet signal, with a small portion of the signal diffusively distributed to perinuclear regions and cytosol (Fig. 1C). Treatment with the FAK inhibitor PF-573228 (10 μM, >60 min) significantly reduced the ECFP/FRET ratio from 1.4 to 0.8 in adherent MEFs (*P* < 0.0001; Fig. 1, D and E). This change represents a ~40% decrease in ECFP/FRET ratio, confirming the specificity of the biosensor. Under FAK inhibition, YPet intensity slightly increased (Fig. 1C); these results are in line with prior work that reported impaired FA disassembly in FAK-null cells (*22*, *25*). We further analyzed biosensor activity via fluorescence lifetime imaging microscopy (FLIM) (*26*) by mapping changes in ECFP fluorescence lifetime (average time in excited state). When FRET is not occurring, the donor fluorescence lifetime becomes longer. Unlike intensity-based FRET measurements, FLIM is not confounded by fluorophore concentration or excitation light intensity (*27*–*29*). In our study, FLIM revealed a longer distribution of fluorescence lifetimes for the FAK biosensor at FAs (2.28 ± 0.06 ns, Fig. 1F) compared to fluorescence lifetimes for the biosensor in the cytosol (2.13 ± 0.04 ns). As expected, cells expressing an ECFP-only (without YPet) construct displayed significantly longer lifetimes (2.58 ± 0.08 ns). Last, lower FRET efficiencies were detected for the FAK biosensor localized to FAs compared to biosensor in the cytosol (Fig. 1G). In summary, the FRET and FLIM analyses confirm the ability of the FAK biosensor to report on real-time FAK activity at FAs in living cells.

### Spatiotemporal patterns of traction force and FAK activity during cell migration

Cell migration involves a tightly coordinated cycle of protrusion formation via actin polymerization, FA assembly, actomyosin contractility, and FA disassembly (*14*, *30*–*33*). FAs assemble at the leading edge, whereas FAs disassemble at the rear and at the base of protrusions, to enable cell motility (*31*). In our prior work, we demonstrated a linear relationship between the magnitudes of traction forces and FAK Y397 phosphorylation at individual FAs on stiff (14 kPa) but not on soft (5 kPa) substrates (*13*). Here, we expand on these findings by exploring the spatiotemporal coordination of traction force and FAK activity during cell migration.

**Fig. 2.**
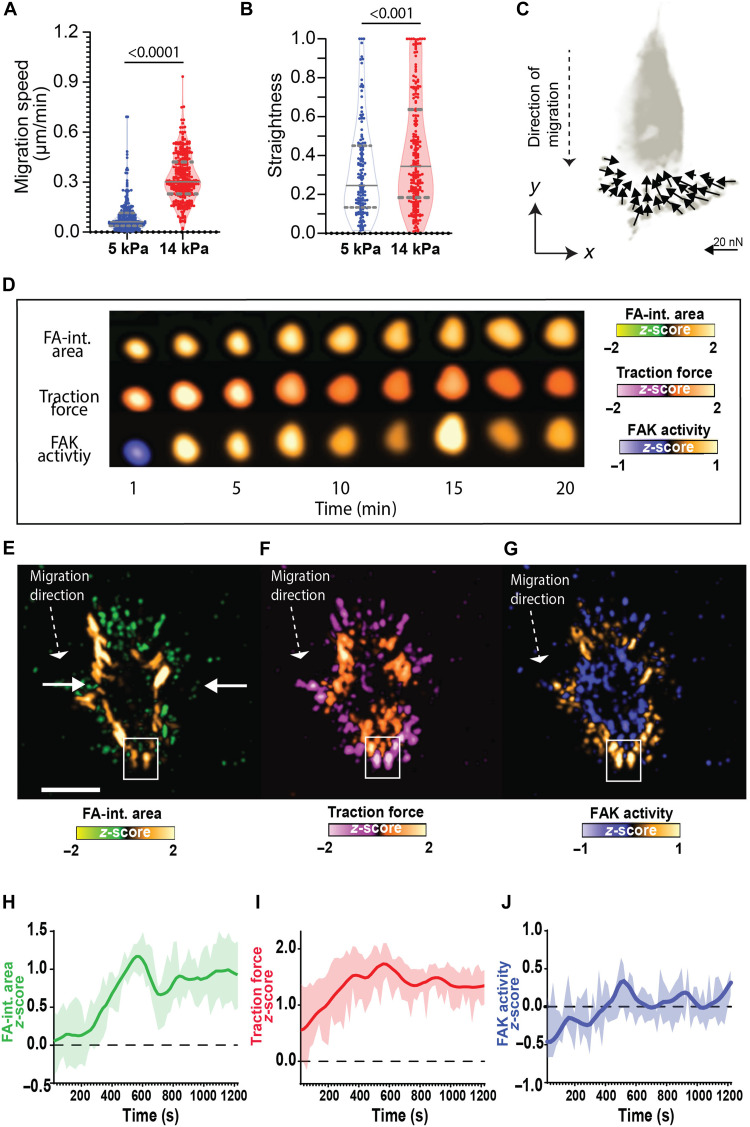
Asymmetric spatial distribution of traction forces and FAK activity in FAs at leading edge correlates with the direction of cell motion. Violin plots depicting (**A**) cell migration speeds and (**B**) straightness over time for cells migrating on soft (5 kPa) and stiff (14 kPa) substrates. The solid line within each violin represents the median speed, while dashed lines indicate the first and third quartiles. Statistical differences between groups were assessed using Mann-Whitney tests. (**C**) Polarized cell illustrating lamellipodium and traction force generation at the leading edge. Arrows indicate direction and magnitude of traction force at individual FAs. (**D**) High magnification of an individual growing FA at the leading edge of a cell. Centered and time-averaged time lapse images representing mean distribution of (**E**) FA-integrated (int.) area, (**F**) traction force, and (**G**) FAK activity in a migrating cell. The migratory direction is indicated by a dashed arrow. Scale bar, 20 μm. Pseudocolor scales represent normalized *z*-scores. Temporal profiles of FAs at the leading edge (white square) showing differences in (**H**) FA-integrated area, (**I**) traction force, and (**J**) FAK activity. Data represent *n* = 58 FAs analyzed from 11 cells across three independent experiments.

To explore the spatiotemporal relationship between traction force and FAK activity during cell migration, we cultured MEFs expressing the FAK-FAT biosensor on fibronectin-coated micropillar array detectors (mPADs) with different substrate stiffness (5 kPa versus 14 kPa) and monitored cell motility over a 20-hour period (fig. S2). Cells exhibit significantly higher speed on stiff mPADs compared to soft substrates (*P* < 0.0001; stiff, 0.32 μm/min; soft, 0.11 μm/min; Fig. 2A). Furthermore, cells on stiff substrates display more persistent motion (straightness index stiff, 0.42; soft, 0.32; *P* < 0.0010; Fig. 2B). FAK-null cells on stiff substrates display lower migration speeds than FAK-expressing cells and similar to those observed in FAK-expressing cells on soft substrates (FAK wild type, 0.35 μm/min; FAK null, 0.18 μm/min; *P* < 0.0001; fig. S3), consistent with prior reports that FAK activity directs migratory behavior on stiff versus soft matrices (*25*, *34*).

A substantial advantage of mPAD-based traction force microscopy over traditional methods using bulk gels is that each micropillar deflection is mechanically independent from the surrounding micropillars, allowing isolation of forces and signaling events at the FA of interest (Fig. 2C). We developed specialized MATLAB code to track and analyze micropillar deflection, ECFP/FRET signal, and YPet signal for spatiotemporal analyses at individual FAs (movies S1 to S4). Briefly, the YPet-tagged images were preprocessed using bleach correction and Laplacian of Gaussian filtering. Automatic binarization, watershed, and morphological segmentation were performed to define FAs. This approach allows for accurate identification, segmentation, and analysis of FAs based on their morphological and fluorescence characteristics. Last, FA signals were averaged over individual FA area to ensure sufficient statistical power and obtain one median value for each FA.

To analyze dynamic events at FAs, we constructed spatial heat maps of integrated FA area (YPet signal), traction force magnitude (micropillar deflection), and FAK activity (ECFP/FRET signal, Fig. 2D) using temporal *z*-score normalization, which standardizes data for all FAs in a cell across time by centering the mean at zero and scaling the SD to one (fig. S4, A and B). *z*-score normalization allows for (i) direct intermetric comparisons, (ii) time-based correlation analyses, and (iii) detection of biologically relevant changes from background noise. To allow for quantitative comparisons among migrating cells, we applied a cell position normalization scheme that recentered the cell body during a 10-min period of persistent migration to the same reference coordinates (fig. S5). This approach enabled time averaging of the recentered image sequences to reveal spatial patterns of FA-integrated area, traction force, and FAK activity during migration (Fig. 2, E to G, and fig. S5). Using this method, we observed clearly defined FA spatial patterns, where larger FAs and low traction forces predominantly localized at the sides of the cell, whereas high traction forces and FAK activity localized to newly forming adhesions at the leading edge. In contrast, the rear of the cell exhibited smaller FAs with reduced force and FAK activity, reflecting the coordinated processes of FA disassembly during migration. Quantitative analysis of the spatial distribution of traction forces at the leading edge revealed a strong correlation with FAK activity (correlation coefficient, 0.86), highlighting a polarized pattern that directs cell migration (fig. S5). This correlation contrasts with that for the FA-integrated area, which showed lower correlation with FAK activity (correlation coefficient, 0.26). Overall, these patterns show the spatial coordination of traction force and FAK activity that facilitate directional movement.

Next, we investigated how traction force and FAK activity evolve over time at FAs at the leading edge during cell migration. During protrusion formation, nascent FAs assemble within the leading edge driven by actin polymerization (*33*). These nascent adhesions then mature through myosin-mediated tension (*6*), which transforms them from small circular FAs into larger plaques. The forces arising from protrusion dynamics determine the direction of cell motion (*21*, *31*, *35*). This process is accompanied by FAK phosphorylation and vinculin recruitment, indicating structural reinforcement and stabilization (*36*, *37*). Consistent with this model, we observed that FAs at the leading edge (white square, Fig. 2, E to G) grew rapidly in area as well as traction force, increasing rapidly for 6 to 9 min until reaching a plateau (Fig. 2, H and I). In contrast, FAK activity remained low (negative *z*-score) initially and increased steadily to elevated values after traction force and FA-integrated area had reached peak values (Fig. 2J).

### Coupled traction force and FAK activity at growing FAs

**Fig. 3.**
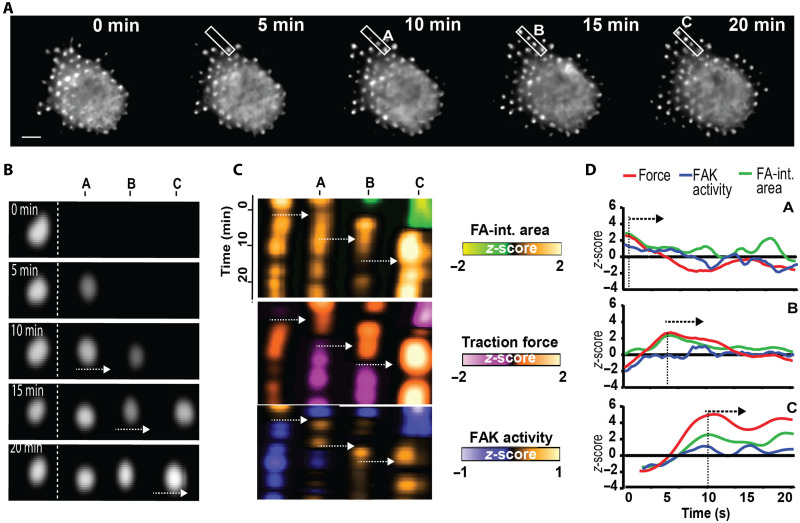
Growing FAs display coordinated mechanochemical activity. (**A**) Time-lapse YPet images showing changes in cell area and FA turnover during migration. Scale bar, 10 μm. (**B**) Single-FA spatiotemporal tracking showing the sequential assembly of FAs during protrusion formation (A, B, and C). (**C**) Kymographs of newly assembled protrusive FAs (A, B, and C) showing FA-integrated (int.) area (top), traction force (middle), and FAK activity (bottom), illustrating the coordinated mechanochemical activity over a 20-min period. (**D**) Corresponding temporal profiles of FA-integrated area, traction force, and FAK activity. *z*-score values represent individual FA traces, shown as representative examples.

Prior work has shown that downregulation of force-transmitting proteins significantly decreases directionality in migration (*35*, *38*–*40*), suggesting spatiotemporal coordination of traction force and FAK activity at FAs. We posit that this spatiotemporal coupling is propagated between adjacent FAs, enhancing the efficiency of force transduction along the axis of force application. To better understand how single FAs dynamics lead to directional migration, we tracked adjacent FAs during protrusion formation at the leading edge of migrating cells (Fig. 3A). We observed coordinated FAK activity and traction forces among adjacent FAs. Protrusive FAs grew in a coordinated wave-like manner, one after another (Fig. 3B), and, notably, FAK activity and traction force propagated sequentially through the newly assembled protrusive FAs (Fig. 3, C and D). This synchronized, dynamic behavior resembles myosin-mediated lamellipodia growth (*31*, *41*) and suggests that both spatial and temporal coordination of mechanochemical activity are essential for efficient directional migration.

**Fig. 4.**
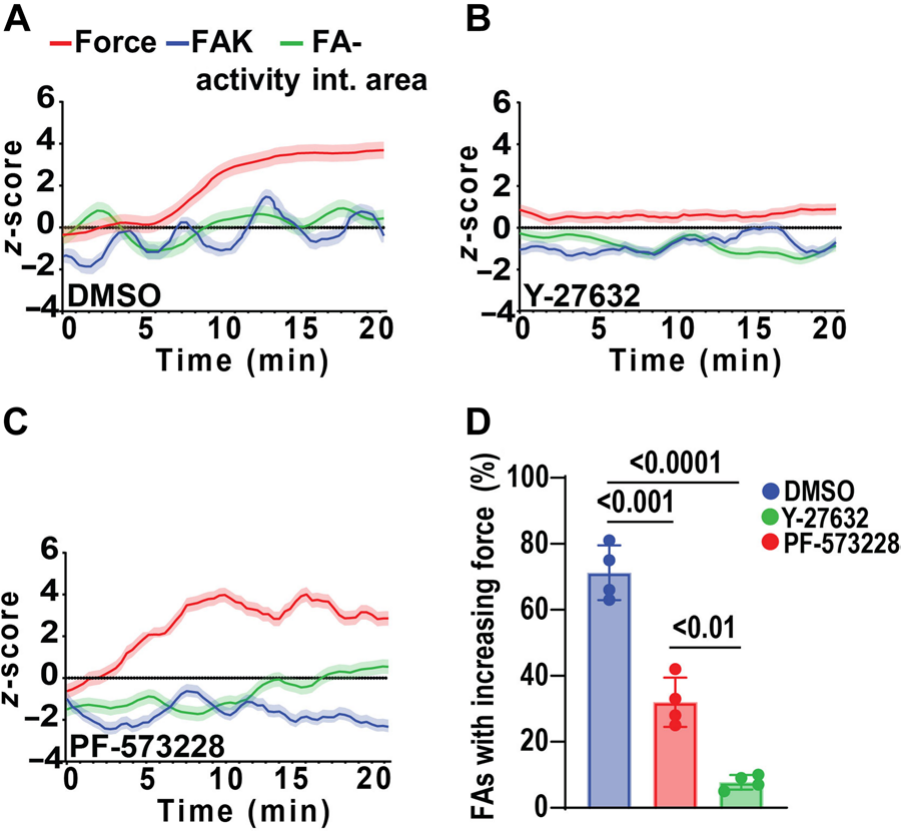
Traction force–FAK activity coupling at FAs requires actomyosin contractility. Temporal profiles of traction force, FAK activity, and FA-integrated (int.) area for (**A**) dimethyl sulfoxide (DMSO), (**B**) Y-27632, and (**C**) PF-573228. (**D**) Proportion of FAs with increasing or steady traction forces in cells treated with DMSO (control), PF-573228, or Y-27632. Data represent *n* = 99 FAs for nine cells replicated in four independent experiments.

We evaluated the dependence of traction force–FAK activity coupling at FAs on actomyosin contractility and FAK activity. Upon contractility inhibition with the ROCK inhibitor Y-27632 (10 μM, 120 min), traction forces and FAK activity did not increase over time and remained at background levels compared to control cells (Fig. 4, A and B), showing that contractility is required for traction force and FAK activity at growing FAs. FA-integrated area was not affected by ROCK inhibition, consistent with prior observations that ROCK inhibition does not prevent FA growth (*42*). Treatment with Y-27632 significantly reduced the proportion of FAs with increasing or high traction force at the leading edge and exhibiting mechanochemical activity (*P* < 0.0001, Fig. 4D). In contrast, treatment with the FAK inhibitor PF-573228 (10 μM for 60 min) did not alter the temporal increases in traction force or FA-integrated area growth (Fig. 4C). As expected, FAK activity remained low (negative *z*-score) upon PF-573228 treatment. Treatment with PF-573228 also reduced the proportion of active FAs compared to control (*P* < 0.0100) but to a lesser extent than for contractility inhibition (*P* < 0.0010, Fig. 4D). This result shows that FAK inhibition suppresses sustained activation but does not eliminate the mechanochemical responses associated with force generation.

**Fig. 5.**
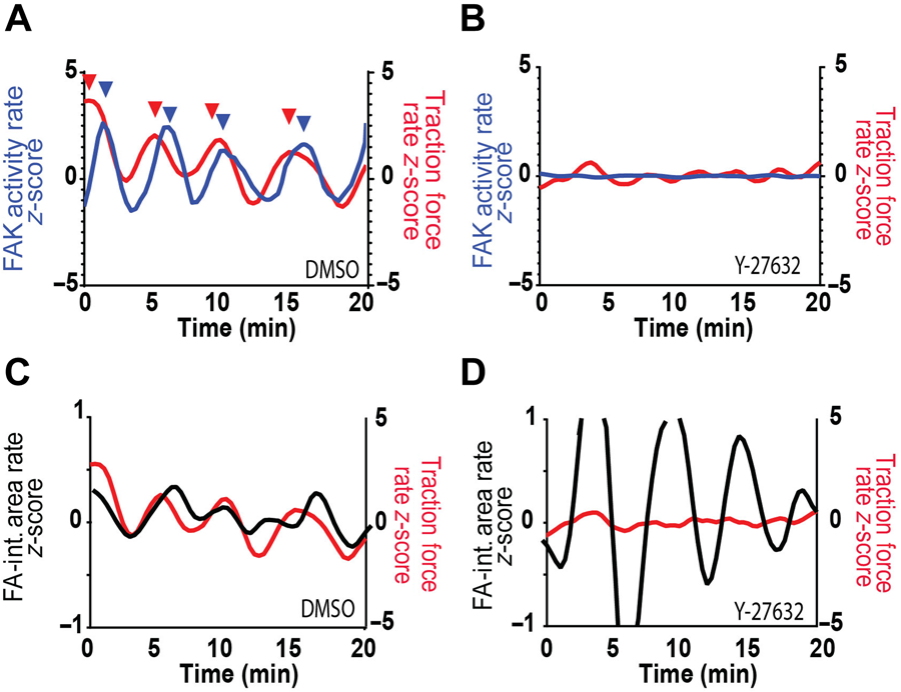
Traction force precedes FAK activity at FAs. (**A**) Temporal profile showing the rates of change in FAK activity and traction force, normalized as *z*-scores for control (DMSO) and (**B**) ROCK inhibition, demonstrating a significant reduction in both traction force and FAK activity. (**C**) Temporal profiles showing the rates of change in FA-integrated (int.) area and traction force for control (DMSO) and (**D**) ROCK inhibition, revealing increased fluctuations in FA-integrated area.

We next analyzed dynamic patterns within single FAs by calculating the *z*-scores for the rates of change for traction force, FAK activity, and FA-integrated area (fig. S4, C and D). Notably, temporal profiles reveal synchronized oscillations for the rates of traction force and FAK activity (Fig. 5A). We refer to these patterns as mechanochemical waves. These oscillatory patterns were abrogated upon treatment with Y-27632 (Fig. 5B). Autocorrelation analyses revealed a distinct periodicity (~5 min) in the rate of traction forces (fig. S6A), which was disrupted with Y-27632 treatment and, to a lesser extent, with PF-573228 inhibition (fig. S6, B and C). Furthermore, cross-correlation analyses demonstrated that, in control cells [dimethyl sulfoxide (DMSO)], traction force rate preceded FAK activity rate (fig. S6D), establishing a clear temporal sequence in mechanochemical waves. This relationship was disrupted with either ROCK or FAK inhibition (fig. S6, E and F), indicating that both actomyosin contractility and FAK catalytic activity are required for the coupling of mechanical forces to FAK signaling at FAs. The rate of FA-integrated area exhibited undulating changes, but these were not in sync with the traction force wave (Fig. 5C). Treatment with Y-27632 ROCK inhibition yielded increased fluctuations in the rate of FA-integrated area (Fig. 5D).

To further analyze the temporal relationship among traction force, FAK activity, and FA-integrated area, we quantified pairwise cross-correlations and extracted both correlation coefficients and lag times across all treatment conditions (DMSO, Y-27632, and PF-573228). In DMSO-treated cells, we observed strong cross-correlation coefficients between all signal pairs, with the highest correlation between force and FA area [correlation coefficient (*r*) = 0.67], followed by force and FAK activity (*r* = 0.61) and FAK and FA area (*r* = 0.49; fig. S6G). Treatment with either FAK or ROCK inhibitors significantly reduced these correlation strengths, indicating that both catalytic activity and contractility are required to sustain synchronized mechanochemical signaling at FAs.

Analysis of lag times revealed that, in DMSO-treated cells, traction force dynamics preceded changes in FAK activity by an average of 32 s (*P* < 0.0001), while FAK activation changes preceded FA area growth by ~21 s (*P* = 0.0494, fig. S6H). The resulting delay between force and FA area was smaller (~10 s on average) and not statistically significant, suggesting that FA area growth becomes rapidly synchronized with traction force once upstream signaling is initiated. These sequential delays support a directional flow of information, and force sensing triggers FAK activation, which, in turn, promotes FA maturation. These temporal relationships were eliminated in cells treated with Y-27632 or PF-573228, confirming that both contractility and FAK activity are required to establish and maintain these mechanochemical oscillations. Overall, these analyses reveal synchronized oscillations of traction force and FAK activity at growing FAs at the leading edge of migrating cells, with the traction force wave driving FAK activity and FA area fluctuations.

### Force releases FAK autoinhibitory FERM-kinase domain interactions

**Fig. 6.**
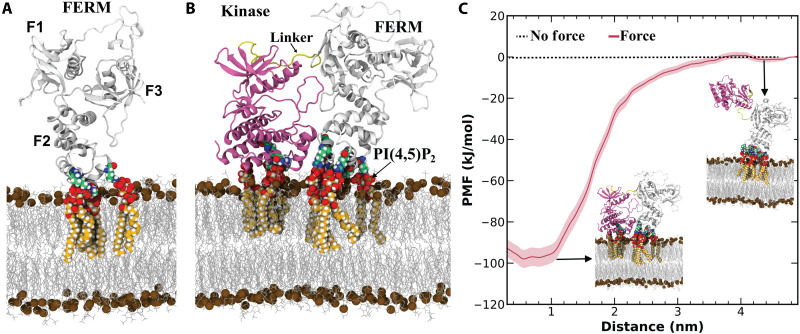
Force is required for FERM-kinase domain dissociation for FAK activation. (**A**) MD simulations showing FAK biding to the membrane. FERM domain bound to membrane POPC/CHOL/SAPS/PI(4,5)P_2_ lipids is stable and does not dissociate or rearrange spontaneously. (**B**) Kinase-FERM domain structural orientation remains stable when bound to membrane. Arg/Lys residues within 5 Å of PI(4,5)P_2_ lipids are shown. For clarity, water, ions and PI(4,5)P_2_ lipids from the lower leaflet are not shown. (**C**) PMF depicting the free energy of binding between the kinase domain and the FERM/membrane as a function of their distance. The kinase domain was pulled diagonally relative to the FERM domain.

Our results support a model in which traction forces drive FAK activation at FAs at the leading edge. To provide further insights into this mechanosignaling switch, we investigated the behavior of the FAK molecule under force using atomistic umbrella sampling (US) molecular dynamics (MD) simulations (Fig. 6A). The resulting potential of the mean force (PMF) reveals a tension-mediated mechanism for the structural separation of the membrane-bound N-terminal FERM domain and the C-terminal FAT domain, which connects to the actin cytoskeleton through paxillin binding (Fig. 6B). These simulations reveal a force threshold for exposure of the kinase domain from the membrane-bound FERM domain. A binding free energy threshold (PMF > 100 kJ/mol) is required at the FAT domain to induce FERM-kinase domain separation and is independent of the force direction or velocity. The structural integrity of these FAK domains is maintained because of the resistance against unfolding of the kinase domain and PIP2 binding sites. Our modeling proposes sequential domain separation and extension, with the FERM and kinase domains initially separated by a substantial distance (>5 nm), followed by the stretching of the interlinking region containing the Y397 phosphorylation site. This separation and stretching create an energetic barrier protecting the kinase domain from unfolding under force, with the FERM domain unfolding only after substantial extension, and even then, PI(4,5)P_2_ binding capability is preserved, acting as a built-in “safety margin.” This ensures that, under typical force levels found within FAs, FAK remains stable and can retain its function. Experimental evidence from single-molecule force spectroscopy supports this model, demonstrating that relatively high mechanical tension can switch FAK from an inhibited to an activated state without fully unraveling its structure (*7*).

## DISCUSSION

We combined micropillar-based traction force microscopy with a FRET-based biosensor to characterize the dynamics of integrin-mediated traction forces and FAK activity at individual FAs during cell migration. Unexpectedly, we found that growing FAs generating high forces at the leading edge exhibit synchronized oscillations of traction force and FAK activity, and cross-correlation analyses demonstrated that force peaks precede increases in FAK activity by ~30 to 60 s. We found that these mechanochemical waves were disrupted by inhibiting contractility or FAK activity, confirming the critical dependence between myosin-mediated traction force generation and FAK catalytic function.

We used a FAK-FRET biosensor to measure FAK activity during cell migration. We validated this FAK biosensor using both ratiometric FRET and FLIM analyses, which exhibited consistent responses. However, ratiometric FRET signals were used for the FA analyses. Ratiometric FRET can be sensitive to intensity variations; however, by using *z*-score normalization over time, the impact of intensity fluctuations on dynamic measurements is minimized. To further assess whether ratiometric FRET measurements might be biased by FA size or intensity, we examined the correlation between FAK activity and FA-integrated area. We observed only a weak correlation between these variables (figs. S5 and S6), suggesting that the ratiometric FRET signal is not simply reporting on FA size or fluorophore accumulation. High local concentrations of FAK or the biosensor could increase FRET signals due to intermolecular donor-acceptor interactions; this would result in an increase in FRET/ECFP and a corresponding decrease in the ECFP/FRET ratio. However, our data show the opposite trend, an increase in the ECFP/FRET ratio in response to FAK activation, indicating that the signal is driven by conformational changes associated with biosensor phosphorylation rather than nonspecific concentration effects. This supports the interpretation that the biosensor accurately reflects local kinase activity. Furthermore, our lifetime-based FRET analyses (FLIM), which are independent of fluorescence intensity, showed similar spatial differences in FAK activity, providing an orthogonal validation of the ratiometric readout and reinforcing the specificity of the probe.

We observed mechanochemical oscillations and their coordination with traction forces. Our data demonstrate that FAK inhibition markedly reduces the ability to sustain high levels of coordinated mechanochemical activity over time. However, FAK inhibition did not eliminate traction force generation or FA signaling. These residual levels of traction force and FAK signaling are consistent with previous reports concluding that FAK acts primarily as a modulator of adhesion dynamics and signaling amplification, rather than as an absolute requirement for basal force transmission.

Additionally, molecular dynamic simulations demonstrate that applied force disrupts autoinhibitory interactions within FAK without unfolding the kinase domain, providing a mechanistic basis for the force-dependent regulation of FAK activity. Whereas our MD simulations provide mechanistic explanations consistent with experimental observations, we acknowledge that these models are intended to complement, not replace, experimental validation. Bauer *et al*. (*7*) demonstrated that mechanical forces activate FAK by disrupting the FERM-kinase interface. Our work expands on these findings by incorporating a lipid membrane environment enriched in PI(4,5)P_2_, a feature not included in earlier models. Recent structural studies by Lietha and colleagues have also highlighted the importance of membrane interactions in regulating FAK activation under force and highlights how FAK-paxillin-vinculin linkage integrates mechanical forces into FA signaling to regulate FAK activation (*43*). Our simulations suggest that membrane composition introduces an additional mechanical checkpoint, requiring greater force inputs to overcome lipid-mediated stabilization and trigger full FAK activation.

Our findings provide previously unrecognized aspects of FAK function, namely, that traction force at growing FAs induces partial dissociation of the FERM-kinase domain interface, exposing Y397 and facilitating its phosphorylation by kinases such as Src. This mechanism demonstrates FAK’s dual role as signaling and mechanosensitive component, contributing to the coordination of cell movement. Structural studies by Acebrón *et al*. (*8*) provided critical insights into FAK activation, showing that PI(4,5)P_2_-enriched membranes induce activation via steric clashes with the kinase domain, leading to a ~90° rotation and FAK oligomerization. However, their cryo-EM analyses primarily focused on static conformational states and lacked quantification of energetic contributions from membrane interactions. In contrast, Bauer *et al*. (*7*) used single-molecule force spectroscopy and MD simulations to demonstrate a different mechanism by which mechanical forces rupture the FERM-kinase interface at ~25 pN, enabling FAK activation. Their findings suggest a “digital switch” model, where force-induced FERM-kinase domain separation precedes unfolding and full activation under physiological tension. Our study expands on these works by examining the combined effects of mechanical forces and FAK-membrane interactions. Our simulations demonstrate that PI(4,5)P_2_ lipids stabilize FAK-membrane interactions. Additionally, the presence of cholesterol may enhance the binding of the FERM-Kinase domain to PI(4,5)P_2_ by increasing head group accessibility and promoting PI(4,5)P_2_ clustering (*44*). This stabilization increases the energy barrier for FERM-kinase domain separation, requiring significantly higher mechanical inputs to release autoinhibition and trigger activation. Thus, cholesterol-rich membranes impose an additional mechanical checkpoint, ensuring that FAK activation occurs only under sufficient mechanical tension, such as actomyosin-generated forces during wound healing, fibrosis, or cancer invasion.

The oscillatory coupling between traction force and FAK activity with a periodicity of ~5 min represents a previously unrecognized mode of adhesion regulation. Early work by Bongrand and colleagues (*45*) showed that cells initially form transient, fluctuating contacts with adhesive surfaces before stabilizing their adhesions. Although these early fluctuations occur on a much faster timescale, these findings suggest that mechanical oscillations are an inherent feature of the adhesion mechanosensory cascade. Our findings extend this concept by showing that maturing FAs exhibit synchronized fluctuations in traction force and FAK activation during migration. We posit that these synchronized oscillations in traction force and FAK activity optimize FA assembly and disassembly during migration. For instance, Waterman and colleagues evaluated subcellular traction dynamics within mature FAs and found that even seemingly “stable” adhesions exhibit internal force fluctuations (*14*, *46*). Using conventional traction force microscopy, they showed that FAs “tug” at the ECM, acting like repeated centripetal pulls on the substrate. This “tugging” was shown to be tension dependent, influenced by ECM stiffness, and modulated by a FAK/phosphopaxillin/vinculin signaling axis. Their results suggest that these autonomous, out-of-phase traction fluctuations among neighboring FAs enable cells to continually sample and respond to their mechanical environment over a broad range of ECM rigidities. Our findings expand on these observations by providing direct evidence that traction force dynamics are synchronized with FAK activation and actomyosin dynamics. We show that changes in FAK activity follow traction force buildup within tens of seconds and that the periodicity of the oscillations matches those of actomyosin contractions (~5-min period). This is particularly relevant because FAK, along with paxillin and vinculin, forms a signaling axis critical for force-dependent FA strengthening and the integration of mechanical cues with downstream biochemical responses. Recent studies have shown a role for actomyosin dynamics in cell migration, particularly concerning the periodic traction force patterns observed across various cell types. For example, *Dictyostelium discoideum* exhibits different migration modes (fan shaped, oscillatory, and amoeboid) that display characteristic traction force patterns and cytoskeletal distributions. These patterns are determined by wave dynamics of actin and myosin, suggesting that oscillatory mechanisms are integral to cell motility. Additionally, theoretical models have proposed that the interplay between contractile forces and mechanosensitive adhesion bonds can lead to complex cell motility behaviors, including periodic back-and-forth movements (*47*–*50*). However, the extent to which these oscillatory patterns are universal across different cell types and environmental contexts remains to be elucidated. Factors such as substrate stiffness, ECM composition, and the expression levels of cytoskeletal proteins modulate these dynamics. Therefore, further studies should consider the conservation of oscillatory behavior in cell migration and assess how cellular and environmental factors influence these mechanisms.

## MATERIALS AND METHODS

### Cell culture and reagents

FAK^+/+^, FAK^−/−^, and FAK-reconstituted mouse embryonic fibroblasts (MEFs) were used as described (*51*) and originally provided by D. D. Schlaepfer at the University of California, San Diego (UCSD). MEFs were cultured in Dulbecco’s modified Eagle’s medium (DMEM; high glucose, Gibco, catalog no. 11965092) supplemented with 10% fetal bovine serum (Gibco, catalog no. A5256701), 1% sodium pyruvate (Gibco, catalog no. 11360070), and 1% penicillin-streptomycin (Gibco, catalog no. 15140122) at 37°C and 5% CO_2_. Cells were sorted by flow cytometry to ensure equivalent expression levels, maintained under puromycin selection (2 μg/ml; Gibco catalog no. A1113803), confirmed mycoplasma-free (Lonza, catalog no. LT07-318), and used between passages 8 and 15. ROCK inhibition was performed with Y-27632 (10 μM; Sigma-Aldrich, catalog no. Y0503; 120 min) and FAK inhibition with PF-573228 (10 μM; Tocris, catalog no. 3239; RRID:SCR_003689; 60 min). All inhibitors were dissolved in DMSO, and equivalent volumes of DMSO (0.1%) were added to control samples.

### FAK FRET biosensor expression

The FA-targeted FAK-FRET biosensor (*22*) was introduced into MEFs via lentiviral transduction. Briefly, lentiviral particles encoding the FRET biosensor were produced in human embryonic kidney 293T cells using standard second-generation packaging (psPAX2, pMD2.G; Addgene, nos. 12260 and 12259). MEFs were transduced at multiplicity of infection of ~10 in the presence of polybrene (8 μg/ml; Sigma-Aldrich, catalog no. H9268) and selected by flow cytometry. Stable populations were validated by fluorescence microscopy for biosensor localization at FAs (coexpression with mCherry-paxillin) and by responsiveness to FAK inhibition (PF-573228).

### mPAD fabrication

Silicon masters for mPADs were fabricated using polydimethylsiloxane (PDMS) replica molding (*52*, *53*). To fabricate templates, 1:10 PDMS prepolymer (PDMS:curing agent ratio, w/w; Sylgard 184, Dow Corning) was cast on mPAD silicon masters, cured at 110°C for 30 min, peeled, with oxygen-plasma treated (Plasma-Preen; Terra Universal), and silanized overnight with (tridecafluoro-1,1,2,2,-tetra-hydrooctyl)-1-trichlorosilane (Sigma-Aldrich, catalog no. 448931) under vacuum. Final mPAD devices are fabricated by casting 1:10 PDMS prepolymer on salinized silicon masters, degassing (20 min), and curing at 110°C for 20 hours and peeling onto a 25-mm-diameter #1 coverslip (Electron Microscopy Services). Collapse of posts during peeling was rectified by sonication in 100% ethanol and critical point drying in CO_2_ (Samdri-PVT-3D; Tousimis).

For functionalization, flat PDMS stamps were coated with fibronectin [Thermo Fisher Scientific, D307; 50 μg/ml in phosphate-buffered saline (PBS)] and Alexa Fluor 647–labeled fibrinogen (Thermo Fisher Scientific, F35200; 20 μg/ml in PBS) for 1 hour, rinsed with water, nitrogen dried, and stamped onto oxidized mPADs (UVO-Model 342; Jelight). Surfaces were passivated with 0.2% Pluronic F-127 (Sigma-Aldrich, catalog no. P2443) for 30 min to prevent nonspecific binding.

### Traction forces

Micropillar deflections were measured from fluorescent images of posts tops and calculated by comparing the centroids of posts under loaded and unloaded conditions using custom MATLAB scripts (MathWorks, R2023a; RRID:SCR_001622). Traction forces were determined using the Euler-Bernoulli beam equation

$$F=\frac{3\mathrm{EI}\delta }{L^{3}}$$

where *E* is the Young’s modulus of PDMS, *I* is the second moment of area for cylindrical posts, δ is the post deflection, and *L* is the post height.

### Confocal microscopy

Raw images were taken on a Nikon Ti2-E motorized inverted microscope and AX-R confocal system, using a high magnification objective (×40 CFI60 Apochromat Lambda S LWD 40× water immersion objective lens, numerical aperture of 1.15, working distance (W.D.) of 0.59 to 0.61 mm, field of view (FOV) of 22 mm, differential interference contrast, correction collar of 0.15 to 0.19 mm). Poisson noise was reduced using the NIS.ai deep learning–based denoising tool (Nikon NIS-Elements, RRID:SCR_014329), which improved signal-to-noise ratio of ~16-fold and enabled lower illumination power and higher acquisition frequency for FRET imaging. Images were exported as 12-bit TIFF files, and further analysis was performed using ImageJ (RRID:SCR_002285) and MATLAB software (MathWorks, R2023a; RRID:SCR_001622).

### Live-cell imaging on mPADs

Cells were seeded onto mPAD substrates in complete growth medium and allowed to adhere for 5 min. Immediately after seeding, mPAD substrates were transferred to a live-cell imaging chamber (AttoFluor; Invitrogen, catalog no. A7816) and maintained in a temperature- and CO_2_-controlled stage incubator (LiCONiC instruments, STXG SP400 Incubation System) during imaging. Traction force and FAK activity were measured in real time using a combination of mPAD-based force microscopy and FRET imaging.

### FRET imaging

For fast high-resolution FRET imaging, ECFP (464 to 499 nm) and YPet (534 to 599 nm) emissions were acquired using 445- and 514-nm lasers, respectively. mPAD images were acquired using a 640-nm laser (650 to 699 nm). Laser power, gain, and dwell time were optimized using Nikon NIS-Elements (RRID:SCR_014329) to minimize spectral bleed-through and maximize signal-to-noise ratio. Pseudocolor images were generated to visualize the spatial distribution of FAK activity at FAs. FRET efficiency was calculated as the ratio of ECFP to YPet emissions and corrected for spectral bleed-through using single-labeled control samples. Data analysis, including correlation of traction forces with FAK activity at individual FAs, was performed using custom MATLAB scripts (MathWorks, R2023a; RRID:SCR_001622).

### FLIM-FRET

To assess FAK activity at FAs, we used FLIM-FRET microscopy. Cells were excited at 445 nm, and fluorescence lifetime data were collected using a cooled hybrid photomultiplier tube (PicoQuant). The acquired data were analyzed with SymPhoTime software (PicoQuant; RRID:SCR_016186), using a biexponential decay model to calculate weighted mean lifetimes and determine FRET efficiency. Spatial distributions of FAK activity at FAs were visualized through lifetime maps.

### FA segmentation and tracking

FA segmentation was performed in Fiji/ImageJ (RRID:SCR_002285) using a macro with bleach correction (simple ratio method), noise reduction [Laplacian of Gaussian (LoG) followed by three-dimensional median filters], thresholding (triangle method), morphological operations, and watershed separation. This process generated the result image highlighting segmented FAs. Regions of interest (0.2 to 20 μm^2^) were identified and saved for analysis. Tracking of individual FAs was done with TrackMate (*54*) using the Linear Assignment Problem (LAP) tracker, linking objects across frames with a maximum displacement of 2 μm. The tracking algorithm LAP tracker (*55*) was used to link objects across consecutive frames allowing for merging of tracks. Tracks spanning ≥7 consecutive frames were retained; FAs that split or moved excessively were excluded. A normalized FA size index (area × mean brightness) was calculated for each trajectory, with assembling adhesions defined by increases >30% above baseline and disassembling adhesions by decreases >30%.

### Definition of FAs with increasing force

FAs were classified on the basis of traction dynamics by applying linear regression to force traces over a rolling 5-min window. Adhesions with a slope ≥ 0.01 nN/min were defined as “increasing force.”

### FRET image processing

FRET analysis followed established methods (*56*, *57*). Background was removed by Costes automatic thresholding (*58*), and spectral bleed-through coefficients were calculated from single-labeled controls. Corrected FRET (cFRET) intensity was computed as

$$\text{cFRET}=I_{\text{FRET}}-\alpha .I_{\text{ECFP}}-\beta .I_{\text{YPET}}$$

with donor and acceptor constants α = 0.175 and β = 0.056 (*59*). Normalized FRET was defined as donor/cFRET. Pseudocolor lookup tables (LUTs) were applied for visualization. Spectral bleed-through coefficients were confirmed using cells expressing only ECFP or YPet in donor, acceptor, and FRET channels.

### MD simulations of FAK binding to membrane

To investigate the binding of FAK (FERM and kinase domains) with the lipid membrane, we used the crystal structure Protein Data Bank ID: 2J0J (*20*). The missing loops in the structure were modeled using the MODELLER tool (RRID:SCR_008395) (*60*). The membrane patch, composed of 256 1-palmitoyl-2-oleoyl-sn-glycero-3-phosphocholine (POPC), 204 cholesterol (CHOL), 26 1-stearoyl-2-arachidonoyl-sn-glycero-3-phospho-L-serine (SAPS), and 26 1-stearoyl-2-arachidonoyl-sn-glycero-3-phospho-(1’-myo-inositol-4’,5’-bisphosphate) (SAPI) lipids (in a 50:40:5:5 ratio), was prepared using the CHARMM-GUI server (RRID:SCR_014892) (*61*). Here, the phosphatidylinositol (PI) headgroup corresponds to PI(4,5)P_2_. The protein was positioned ~1 nm above the lipid phosphorus (P) atom. The system was solvated with 141,651 CHARMM-modified Transferable Intermolecular Potential with 3 Points (TIP3P) (*62*) water molecules in a box with dimensions of 11.5 nm by 11.5 nm by 17.5 nm in the *x*, *y*, and *z* directions. The total system charge was neutralized by adding 134 K+ ions, resulting in a total of 209,072 atoms. The system was energy minimized using the steepest descent algorithm, following by equilibration for 500 ps with position restraints on the protein backbone atoms. During equilibration, the temperature was regulated at 310 K with the Berendsen thermostat (τ_t_ = 1 ps) (*63*), and the pressure was controlled at 1 bar using the Berendsen semi-isotropic scheme (τ_p_ = 5 ps). A cutoff of 1.2 nm was applied to both Lennard-Jones interactions and electrostatic interactions, with the latter being treated using the particle-mesh Ewald method (*64*). Hydrogen bonds were constrained using the Linear Constraint Solver (LINCS) algorithm (*65*). For the production simulations, all position restraints were removed. The temperature was controlled using the velocity rescaling thermostat (*66*), while the pressure was controlled using the semi-isotropic Parrinello-Rahman barostat (*67*). All other parameters were identical to those used during the equilibration run. The production simulations were conducted for 1 μs in triplicate with an integration time step of 2 fs, using GROMACS 2021 (RRID:SCR_014565) simulation software (*68*). All figures and plots were generated using VMD (RRID:SCR_001820) (*69*) and Matplotlib (RRID:SCR_008624) (*70*).

To assess the stability of the FERM domain on the membrane, we conducted additional simulations by removing the kinase domain from the structure. These simulations were performed in triplicate, each for 1 μs. The membrane patch and simulation parameters were identical to those described above.

### PMFs for kinase domain interacting with FERM/membrane

The last frame from the kinase/FERM-membrane production simulation was used as the initial configuration for the PMF calculations. Because the kinase domain is pulled diagonally relative to the FERM domain, we increased the box dimensions in the *xy* plane by ~2 nm. Thus, the membrane patch comprised 376 POPC, 300 CHOL, 40 SAPS, and 40 SAPI lipids (in a 50:40:5:5 ratio) solvated with 224,442 CHARMM-modified TIP3P (*62*) water molecules in a box with dimensions of 13.5 nm by 13.8 nm by 17.5 nm. The total system charge was neutralized by adding 194 K+ ions, resulting in a total of 318,503 atoms.

To estimate the binding free energy or PMFs for the interaction of the kinase domain with the FERM/membrane, we performed US MD simulations. The reaction coordinate was defined by the distance between the center of mass of the kinase domain and the center of mass of the FERM domain. Initial pulling simulations of the kinase domain were performed over 32 ns with an umbrella potential along the direction defined by a specific vector (pull-geometry: direction, pull-vec: -2.5795 -0.23896 -5.83106, pull-dim: Y Y Y). The kinase domain was pulled at a rate of 0.15 nm/ns with a force constant of 1000 kJ mol^−1^ nm^−2^. FERM-membrane simulations (without the kinase domain) revealed that the FERM domain adopts an upright orientation while binding to the PI(4,5)P_2_ lipids. To maintain the upright orientation of the reference FERM domain during pulling simulations, backbone position restraints were applied in the *x*-*y* directions on a few selected atoms of the FERM domain. The remaining simulation parameters were the same as those described above for the production simulation.

From the pulling simulations, 67 US windows were extracted with a spacing of 0.05 nm until the kinase domain was pulled away by a distance of 2.1 nm from the FERM/membrane. After that, the spacing was set to 0.1 nm. Each US window was simulated for 100 ns with a force constant of 1000 kJ mol^−1^ nm^−2^. The PMFs were computed by skipping the first 20 ns for equilibration, using the weighted histogram analysis method (gmx wham) (*71*, *72*) implemented in the GROMACS 2021 (RRID:SCR_014565) simulation package (*68*). SEs were estimated over 50 rounds of bootstrapping analysis.

### Statistical analysis

Experiments were performed in at least three independent biological replicates. Data are shown as median ± interquartile range. Statistical significance was assessed using Kruskal-Wallis (Mann-Whitney test for two groups) with Dunn’s post hoc comparisons. Analyses were performed using GraphPad Prism (RRID:SCR_002798), and *P* < 0.05 was considered significant.

## Notes

**Correction (16 April 2026):** The original version of the text incorrectly cited the RRID SCR_015528 for TrackMate in the section “FA segmentation and tracking.” This RRID, which belongs to a different software, has been replaced with a citation to the following reference, now (*54*): D. Ershov, M.-S. Phan, J. W. Pylvänäinen, S. U. Rigaud, L. Le Blanc, A. Charles-Orszag, J. R. W. Conway, R. F. Laine, N. H. Roy, D. Bonazzi, G. Duménil, G. Jacquemet, J.-Y. Tinevez, TrackMate 7: integrating state-of-the-art segmentation algorithms into tracking pipelines, *Nat. Methods* **19**, 829–832 (2022); https://www.nature.com/articles/s41592-022-01507-1. Subsequent references were renumbered. The PDF and online versions of the article have been updated.

## References

[1] M. A. Wozniak, C. S. Chen, Mechanotransduction in development: A growing role for contractility. Nat. Rev. Mol. Cell Biol. 10, 34–43 (2009). 19197330 10.1038/nrm2592 PMC2952188

[2] D. E. Discher, D. J. Mooney, P. W. Zandstra, Growth factors, matrices, and forces combine and control stem cells. Science 324, 1673–1677 (2009). 19556500 10.1126/science.1171643 PMC2847855

[3] C. J. Chan, M. Costanzo, T. Ruiz-Herrero, G. Monke, R. J. Petrie, M. Bergert, A. Diz-Munoz, L. Mahadevan, T. Hiiragi, Hydraulic control of mammalian embryo size and cell fate. Nature 571, 112–116 (2019). 31189957 10.1038/s41586-019-1309-x

[4] C. C. DuFort, M. J. Paszek, V. M. Weaver, Balancing forces: Architectural control of mechanotransduction. Nat. Rev. Mol. Cell Biol. 12, 308–319 (2011). 21508987 10.1038/nrm3112 PMC3564968

[5] V. Vogel, M. P. Sheetz, Cell fate regulation by coupling mechanical cycles to biochemical signaling pathways. Curr. Opin. Cell Biol. 21, 38–46 (2009). 19217273 10.1016/j.ceb.2009.01.002 PMC3792581

[6] J. T. Parsons, A. R. Horwitz, M. A. Schwartz, Cell adhesion: Integrating cytoskeletal dynamics and cellular tension. Nat. Rev. Mol. Cell Biol. 11, 633–643 (2010). 20729930 10.1038/nrm2957 PMC2992881

[7] M. S. Bauer, F. Baumann, C. Daday, P. Redondo, E. Durner, M. A. Jobst, L. F. Milles, D. Mercadante, D. A. Pippig, H. E. Gaub, F. Grater, D. Lietha, Structural and mechanistic insights into mechanoactivation of focal adhesion kinase. Proc. Natl. Acad. Sci. U.S.A. 116, 6766–6774 (2019). 30877242 10.1073/pnas.1820567116 PMC6452671

[8] I. Acebrón, R. D. Righetto, C. Schoenherr, S. de Buhr, P. Redondo, J. Culley, C. F. Rodriguez, C. Daday, N. Biyani, O. Llorca, A. Byron, M. Chami, F. Grater, J. Boskovic, M. C. Frame, H. Stahlberg, D. Lietha, Structural basis of focal adhesion kinase activation on lipid membranes. EMBO J. 39, e104743 (2020). 32779739 10.15252/embj.2020104743 PMC7527928

[9] F. A. Herzog, L. Braun, I. Schoen, V. Vogel, Structural insights how PIP2 imposes preferred binding orientations of FAK at lipid membranes. J. Phys. Chem. B 121, 3523–3535 (2017). 28124908 10.1021/acs.jpcb.6b09349

[10] S. M. Weis, S. T. Lim, K. M. Lutu-Fuga, L. A. Barnes, X. L. Chen, J. R. Gothert, T. L. Shen, J. L. Guan, D. D. Schlaepfer, D. A. Cheresh, Compensatory role for Pyk2 during angiogenesis in adult mice lacking endothelial cell FAK. J. Cell Biol. 181, 43–50 (2008). 18391070 10.1083/jcb.200710038 PMC2287283

[11] E. G. Kleinschmidt, D. D. Schlaepfer, Focal adhesion kinase signaling in unexpected places. Curr. Opin. Cell Biol. 45, 24–30 (2017). 28213315 10.1016/j.ceb.2017.01.003 PMC5482783

[12] J. C. Kuo, X. Han, C. T. Hsiao, J. R. Yates III, C. M. Waterman, Analysis of the myosin-II-responsive focal adhesion proteome reveals a role for beta-Pix in negative regulation of focal adhesion maturation. Nat. Cell Biol. 13, 383–393 (2011). 21423176 10.1038/ncb2216 PMC3279191

[13] D. W. Zhou, M. A. Fernandez-Yague, E. N. Holland, A. F. Garcia, N. S. Castro, E. B. O’Neill, J. Eyckmans, C. S. Chen, J. Fu, D. D. Schlaepfer, A. J. Garcia, Force-FAK signaling coupling at individual focal adhesions coordinates mechanosensing and microtissue repair. Nat. Commun. 12, 2359 (2021). 33883558 10.1038/s41467-021-22602-5 PMC8060400

[14] S. V. Plotnikov, A. M. Pasapera, B. Sabass, C. M. Waterman, Force fluctuations within focal adhesions mediate ECM-rigidity sensing to guide directed cell migration. Cell 151, 1513–1527 (2012). 23260139 10.1016/j.cell.2012.11.034 PMC3821979

[15] A. Elosegui-Artola, R. Oria, Y. Chen, A. Kosmalska, C. Perez-Gonzalez, N. Castro, C. Zhu, X. Trepat, P. Roca-Cusachs, Mechanical regulation of a molecular clutch defines force transmission and transduction in response to matrix rigidity. Nat. Cell Biol. 18, 540–548 (2016). 27065098 10.1038/ncb3336

[16] S. K. Mitra, D. A. Hanson, D. D. Schlaepfer, Focal adhesion kinase: In command and control of cell motility. Nat. Rev. Mol. Cell Biol. 6, 56–68 (2005). 15688067 10.1038/nrm1549

[17] K. M. Young, C. A. Reinhart-King, Cellular mechanosignaling for sensing and transducing matrix rigidity. Curr. Opin. Cell Biol. 83, 102208 (2023). 37473514 10.1016/j.ceb.2023.102208 PMC10527818

[18] A. S. Torsoni, S. S. Constancio, W. Nadruz Jr., S. K. Hanks, K. G. Franchini, Focal adhesion kinase is activated and mediates the early hypertrophic response to stretch in cardiac myocytes. Circ. Res. 93, 140–147 (2003). 12805241 10.1161/01.RES.0000081595.25297.1B

[19] J. Seong, A. Tajik, J. Sun, J. L. Guan, M. J. Humphries, S. E. Craig, A. Shekaran, A. J. Garcia, S. Lu, M. Z. Lin, N. Wang, Y. Wang, Distinct biophysical mechanisms of focal adhesion kinase mechanoactivation by different extracellular matrix proteins. Proc. Natl. Acad. Sci. U.S.A. 110, 19372–19377 (2013). 24222685 10.1073/pnas.1307405110 PMC3845171

[20] D. Lietha, X. Cai, D. F. Ceccarelli, Y. Li, M. D. Schaller, M. J. Eck, Structural basis for the autoinhibition of focal adhesion kinase. Cell 129, 1177–1187 (2007). 17574028 10.1016/j.cell.2007.05.041 PMC2077847

[21] P. Kanchanawong, G. Shtengel, A. M. Pasapera, E. B. Ramko, M. W. Davidson, H. F. Hess, C. M. Waterman, Nanoscale architecture of integrin-based cell adhesions. Nature 468, 580–584 (2010). 21107430 10.1038/nature09621 PMC3046339

[22] Y. Q. Wu, K. W. Zhang, J. Seong, J. Fan, S. Chien, Y. X. Wang, S. Y. Lu, In-situ coupling between kinase activities and protein dynamics within single focal adhesions. Sci. Rep. 6, 29377 (2016). 27383747 10.1038/srep29377 PMC4935953

[23] Y. X. Wang, E. L. Botvinick, Y. H. Zhao, M. W. Berns, S. Usami, R. Y. Tsien, S. Chien, Visualizing the mechanical activation of Src. Nature 434, 1040–1045 (2005). 15846350 10.1038/nature03469

[24] J. Y. Seong, M. X. Ouyang, T. Kim, J. Sun, P. C. Wen, S. Y. Lu, Y. Zhuo, N. M. Llewellyn, D. D. Schlaepfer, J. L. Guan, S. Chien, Y. X. Wang, Detection of focal adhesion kinase activation at membrane microdomains by fluorescence resonance energy transfer. Nat. Commun. 2, 406 (2011). 21792185 10.1038/ncomms1414 PMC3373894

[25] D. J. Webb, K. Donais, L. A. Whitmore, S. M. Thomas, C. E. Turner, J. T. Parsons, A. F. Horwitz, FAK-Src signalling through paxillin, ERK and MLCK regulates adhesion disassembly. Nat. Cell Biol. 6, 154–161 (2004). 14743221 10.1038/ncb1094

[26] C. Grashoff, B. D. Hoffman, M. D. Brenner, R. B. Zhou, M. Parsons, M. T. Yang, M. A. McLean, S. G. Sligar, C. S. Chen, T. Ha, M. A. Schwartz, Measuring mechanical tension across vinculin reveals regulation of focal adhesion dynamics. Nature 466, 263–U143 (2010). 20613844 10.1038/nature09198 PMC2901888

[27] F. S. Wouters, P. I. Bastiaens, Fluorescence lifetime imaging of receptor tyrosine kinase activity in cells. Curr. Biol. 9, 1127–1130 (1999). 10531012 10.1016/s0960-9822(99)80484-9

[28] P. Ringer, A. Weißl, A. L. Cost, A. Freikamp, B. Sabass, A. Mehlich, M. Tramier, M. Rief, C. Grashoff, Multiplexing molecular tension sensors reveals piconewton force gradient across talin-1. Nat. Methods 14, 1090–1096 (2017). 28945706 10.1038/nmeth.4431

[29] P. J. Verveer, F. S. Wouters, A. R. Reynolds, P. I. Bastiaens, Quantitative imaging of lateral ErbB1 receptor signal propagation in the plasma membrane. Science 290, 1567–1570 (2000). 11090353 10.1126/science.290.5496.1567

[30] D. A. Lauffenburger, A. F. Horwitz, Cell migration: A physically integrated molecular process. Cell 84, 359–369 (1996). 8608589 10.1016/s0092-8674(00)81280-5

[31] S. Lo Vecchio, R. Thiagarajan, D. Caballero, V. Vigon, L. Navoret, R. Voituriez, D. Riveline, Collective dynamics of focal adhesions regulate direction of cell motion. Cell Syst. 10, 535–542.e4 (2020). 32553185 10.1016/j.cels.2020.05.005

[32] D. H. Kim, D. Wirtz, Focal adhesion size uniquely predicts cell migration. FASEB J. 27, 1351–1361 (2013). 23254340 10.1096/fj.12-220160 PMC3606534

[33] M. L. Gardel, I. C. Schneider, Y. Aratyn-Schaus, C. M. Waterman, Mechanical integration of actin and adhesion dynamics in cell migration. Annu. Rev. Cell Dev. Biol. 26, 315–333 (2010). 19575647 10.1146/annurev.cellbio.011209.122036 PMC4437624

[34] H. B. Wang, M. Dembo, S. K. Hanks, Y. L. Wang, Focal adhesion kinase is involved in mechanosensing during fibroblast migration. Proc. Natl. Acad. Sci. U.S.A. 98, 11295–11300 (2001). 11572981 10.1073/pnas.201201198 PMC58723

[35] A. Ray, O. Lee, Z. Win, R. M. Edwards, P. W. Alford, D. H. Kim, P. P. Provenzano, Anisotropic forces from spatially constrained focal adhesions mediate contact guidance directed cell migration. Nat. Commun. 8, 14923 (2017). 28401884 10.1038/ncomms14923 PMC5394287

[36] N. Q. Balaban, U. S. Schwarz, D. Riveline, P. Goichberg, G. Tzur, I. Sabanay, D. Mahalu, S. Safran, A. Bershadsky, L. Addadi, B. Geiger, Force and focal adhesion assembly: A close relationship studied using elastic micropatterned substrates. Nat. Cell Biol. 3, 466–472 (2001). 11331874 10.1038/35074532

[37] J. L. Tan, J. Tien, D. M. Pirone, D. S. Gray, K. Bhadriraju, C. S. Chen, Cells lying on a bed of microneedles: An approach to isolate mechanical force. Proc. Natl. Acad. Sci. U.S.A. 100, 1484–1489 (2003). 12552122 10.1073/pnas.0235407100 PMC149857

[38] S. Chen, M. J. Hourwitz, L. Campanello, J. T. Fourkas, W. Losert, C. A. Parent, Actin cytoskeleton and focal adhesions regulate the biased migration of breast cancer cells on nanoscale asymmetric sawteeth. ACS Nano 13, 1454–1468 (2019). 30707556 10.1021/acsnano.8b07140 PMC7159974

[39] G. R. Ramirez-San Juan, P. W. Oakes, M. L. Gardel, Contact guidance requires spatial control of leading-edge protrusion. Mol. Biol. Cell 28, 1043–1053 (2017). 28228548 10.1091/mbc.E16-11-0769 PMC5391181

[40] I. Thievessen, N. Fakhri, J. Steinwachs, V. Kraus, R. S. McIsaac, L. Gao, B. C. Chen, M. A. Baird, M. W. Davidson, E. Betzig, R. Oldenbourg, C. M. Waterman, B. Fabry, Vinculin is required for cell polarization, migration, and extracellular matrix remodeling in 3D collagen. FASEB J. 29, 4555–4567 (2015). 26195589 10.1096/fj.14-268235 PMC4608908

[41] G. Giannone, B. J. Dubin-Thaler, H. G. Dobereiner, N. Kieffer, A. R. Bresnick, M. P. Sheetz, Periodic lamellipodial contractions correlate with rearward actin waves. Cell 116, 431–443 (2004). 15016377 10.1016/s0092-8674(04)00058-3

[42] J. Stricker, Y. Beckham, M. W. Davidson, M. L. Gardel, Myosin II-mediated focal adhesion maturation is tension insensitive. PLOS ONE 8, e70652 (2013). 23923013 10.1371/journal.pone.0070652 PMC3726642

[43] K. Diaz-Palacios, P. Lopez Navajas, B. Rodrigo Martin, R. Matesanz, J. R. Luque-Ortega, A. Echarri, D. Lietha, Phospho-regulated tethering of focal adhesion kinase to vinculin links force transduction to focal adhesion signaling. Cell Commun. Signal 23, 190 (2025). 40259376 10.1186/s12964-025-02201-3 PMC12013189

[44] F. Lolicato, R. Saleppico, A. Griffo, A. Meyer, F. Scollo, B. Pokrandt, H. M. Müller, H. Ewers, H. Hähl, J. B. Fleury, R. Seemann, M. Hof, B. Brügger, K. Jacobs, I. Vattulainen, W. Nickel, Cholesterol promotes clustering of PI(4,5)P_2_ driving unconventional secretion of FGF2. J. Cell Biol. 221, e202106123 (2022). 36173379 10.1083/jcb.202106123 PMC9526255

[45] A. Pierres, A. M. Benoliel, D. Touchard, P. Bongrand, How cells tiptoe on adhesive surfaces before sticking. Biophys. J. 94, 4114–4122 (2008). 18234815 10.1529/biophysj.107.125278 PMC2367202

[46] K. S. Honasoge, Z. Karagoz, B. T. Goult, H. Wolfenson, V. L. S. LaPointe, A. Carlier, Force-dependent focal adhesion assembly and disassembly: A computational study. PLOS Comput. Biol. 19, e1011500 (2023). 37801464 10.1371/journal.pcbi.1011500 PMC10584152

[47] E. Ghabache, Y. Cao, Y. Miao, A. Groisman, P. N. Devreotes, W. J. Rappel, Coupling traction force patterns and actomyosin wave dynamics reveals mechanics of cell motion. Mol. Syst. Biol. 17, e10505 (2021). 34898015 10.15252/msb.202110505 PMC8666840

[48] A. Valencia-Exposito, I. Grosheva, D. G. Miguez, A. Gonzalez-Reyes, M. D. Martin-Bermudo, Myosin light-chain phosphatase regulates basal actomyosin oscillations during morphogenesis. Nat. Commun. 7, 10746 (2016). 26888436 10.1038/ncomms10746 PMC4759631

[49] S. Zhang, X. Teng, Y. Toyama, T. E. Saunders, Periodic oscillations of myosin-II mechanically proofread cell-cell connections to ensure robust formation of the cardiac vessel. Curr. Biol. 30, 3364–3377.e4 (2020). 32679105 10.1016/j.cub.2020.06.041

[50] L. He, X. Wang, H. L. Tang, D. J. Montell, Tissue elongation requires oscillating contractions of a basal actomyosin network. Nat. Cell Biol. 12, 1133–1142 (2010). 21102441 10.1038/ncb2124 PMC3056411

[51] D. A. Hsia, S. T. Lim, J. A. Bernard-Trifilo, S. K. Mitra, S. Tanaka, J. den Hertog, D. N. Streblow, D. Ilic, M. H. Ginsberg, D. D. Schlaepfer, Integrin α4β1 promotes focal adhesion kinase-independent cell motility via α4 cytoplasmic domain-specific activation of c-Src. Mol. Cell. Biol. 25, 9700–9712 (2005). 16227616 10.1128/MCB.25.21.9700-9712.2005 PMC1265817

[52] D. W. Dumbauld, T. T. Lee, A. Singh, J. Scrimgeour, C. A. Gersbach, E. A. Zamir, J. Fu, C. S. Chen, J. E. Curtis, S. W. Craig, A. J. Garcia, How vinculin regulates force transmission. Proc. Natl. Acad. Sci. U.S.A. 110, 9788–9793 (2013). 23716647 10.1073/pnas.1216209110 PMC3683711

[53] M. T. Yang, J. Fu, Y. K. Wang, R. A. Desai, C. S. Chen, Assaying stem cell mechanobiology on microfabricated elastomeric substrates with geometrically modulated rigidity. Nat. Protoc. 6, 187–213 (2011). 21293460 10.1038/nprot.2010.189 PMC7183577

[54] D. Ershov, M.-S. Phan, J. W. Pylvänäinen, S. U. Rigaud, L. Le Blanc, A. Charles-Orszag, J. R. W. Conway, R. F. Laine, N. H. Roy, D. Bonazzi, G. Duménil, G. Jacquemet, J.-Y. Tinevez, TrackMate 7: integrating state-of-the-art segmentation algorithms into tracking pipelines, Nat. Methods 19, 829–832 (2022) https://www.nature.com/articles/s41592-022-01507-1. 35654950 10.1038/s41592-022-01507-1

[55] K. Jaqaman, D. Loerke, M. Mettlen, H. Kuwata, S. Grinstein, S. L. Schmid, G. Danuser, Robust single-particle tracking in live-cell time-lapse sequences. Nat. Methods 5, 695–702 (2008). 18641657 10.1038/nmeth.1237 PMC2747604

[56] J. A. Broussard, B. Rappaz, D. J. Webb, C. M. Brown, Fluorescence resonance energy transfer microscopy as demonstrated by measuring the activation of the serine/threonine kinase Akt. Nat. Protoc. 8, 265–281 (2013). 23306460 10.1038/nprot.2012.147 PMC3756929

[57] D. Spiering, J. J. Bravo-Cordero, Y. Moshfegh, V. Miskolci, L. Hodgson, Quantitative ratiometric imaging of FRET-biosensors in living cells. Methods Cell Biol. 114, 593–609 (2013). 23931524 10.1016/B978-0-12-407761-4.00025-7 PMC3789067

[58] S. V. Costes, D. Daelemans, E. H. Cho, Z. Dobbin, G. Pavlakis, S. Lockett, Automatic and quantitative measurement of protein-protein colocalization in live cells. Biophys. J. 86, 3993–4003 (2004). 15189895 10.1529/biophysj.103.038422 PMC1304300

[59] Z. P. Xia, Y. H. Liu, Reliable and global measurement of fluorescence resonance energy transfer using fluorescence microscopes. Biophys. J. 81, 2395–2402 (2001). 11566809 10.1016/S0006-3495(01)75886-9 PMC1301710

[60] N. Eswar, B. Webb, M. A. Marti-Renom, M. S. Madhusudhan, D. Eramian, M.-Y. Shen, U. Pieper, A. Sali, Comparative protein structure modeling using MODELLER. Curr. Protoc. Bioinformatics 10.1002/0471140864.ps0209s50 (2007). 10.1002/0471250953.bi0506s15 PMC4186674 18428767

[61] E. L. Wu, X. Cheng, S. Jo, H. Rui, K. C. Song, E. M. Dávila-Contreras, Y. Qi, J. Lee, V. Monje-Galvan, R. M. Venable, J. B. Klauda, W. Im, CHARMM-GUI membrane builder toward realistic biological membrane simulations. J. Comput. Chem. 35, 1997–2004 (2014). 25130509 10.1002/jcc.23702 PMC4165794

[62] W. L. Jorgensen, J. Chandrasekhar, J. D. Madura, R. W. Impey, M. L. Klein, Comparison of simple potential functions for simulating liquid water. J. Chem. Phys. 79, 926–935 (1983).

[63] H. J. C. Berendsen, J. P. M. Postma, W. F. van Gunsteren, A. DiNola, J. R. Haak, Molecular dynamics with coupling to an external bath. J. Chem. Phys. 81, 3684–3690 (1984).

[64] T. Darden, D. York, L. Pedersen, Particle mesh Ewald: An Nlog(N) method for Ewald sums in large systems. J. Chem. Phys. 98, 10089 (1998).

[65] B. Hess, H. Bekker, H. J. C. Berendsen, J. G. E. M. Fraaije, LINCS: A linear constraint solver for molecular simulations. J. Comput. Chem. 18, 1463–1472 (1997).

[66] G. Bussi, D. Donadio, M. Parrinello, Canonical sampling through velocity rescaling. J. Chem. Phys. 126, 014101–014107 (2007). 17212484 10.1063/1.2408420

[67] M. Parrinello, Polymorphic transitions in single crystals: A new molecular dynamics method. J. Appl. Phys. 52, 7182–7190 (1981).

[68] M. J. Abraham, T. Murtola, R. Schulz, S. Páll, J. C. Smith, B. Hess, E. Lindah, GROMACS: High performance molecular simulations through multi-level parallelism from laptops to supercomputers. SoftwareX 1-2, 19–25 (2015).

[69] W. Humphrey, A. Dalke, K. Schulten, VMD: Visual molecular dynamics. J. Mol. Graph. 14, 33–38 (1996). 8744570 10.1016/0263-7855(96)00018-5

[70] J. D. Hunter, Matplotlib: A 2D graphics environment. Comput. Sci. Eng. 9, 90–95 (2007).

[71] J. S. Hub, B. L. De Groot, D. Van Der Spoel, G-whams—A free weighted histogram analysis implementation including robust error and autocorrelation estimates. J. Chem. Theory Comput. 6, 3713–3720 (2010).

[72] S. Kumar, D. Bouzida, R. H. Swendsen, P. A. Kollman, J. M. Rosenberg, The weighted histogram analysis method for free-energy calculations on biomolecules. 1: The method. J. Comput. Chem. 13, 1011–1021 (1992).

