# Supplementary material for: Mechanochemical waves in focal adhesions during cell migration
Source: Sci Adv. 2025 Oct 3;11(40):eadw6425. doi: 10.1126/sciadv.adw6425 (PMC12494029; doi:10.1126/sciadv.adw6425)
Supplement: Supplementary file 1 — Figs. S1 to S6 Legends for movies S1 to S4 [file sciadv.adw6425_sm.pdf]

Science Advances
AAAS

Supplementary Materials for

Mechanochemical waves in focal adhesions during cell migration

Marc A. Fernández-Yagüe *et al.*

Corresponding author: Andrés J. García, andres.garcia@me.gatech.edu

Sci. Adv. **11**, eadw6425 (2025)
DOI: 10.1126/sciadv.adw6425

The PDF file includes:

Figs. S1 to S6
Legends for movies S1 to S4

Other Supplementary Material for this manuscript includes the following:

Movies S1 to S4

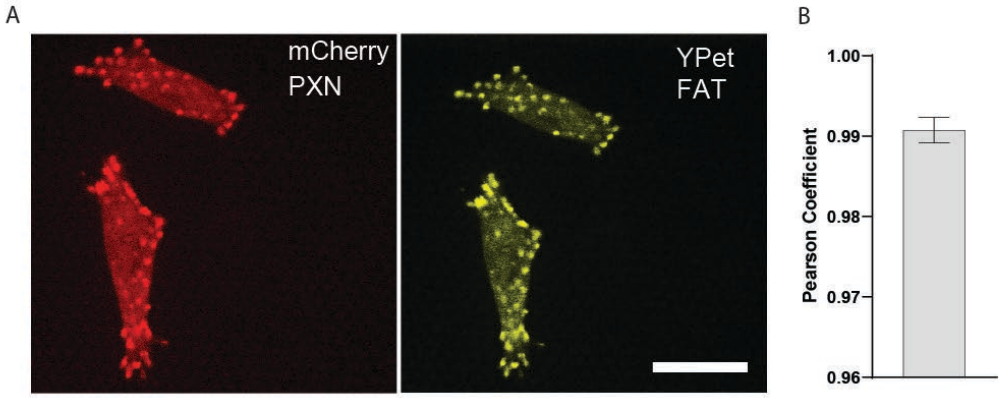

Fig. S1. Correlation analysis between YPet and mChery-paxillin localization. (A) Cells co-expressing the FAK biosensor and FA protein mCherry-paxillin were analyzed for co-localization of YPet and mCherry signals. (**B**) High spatial correlation between YPet and mCherry (Pearson coefficient 0.988 ± 0.001) signals was detected, confirming co-localization of FAK biosensor with paxillin.

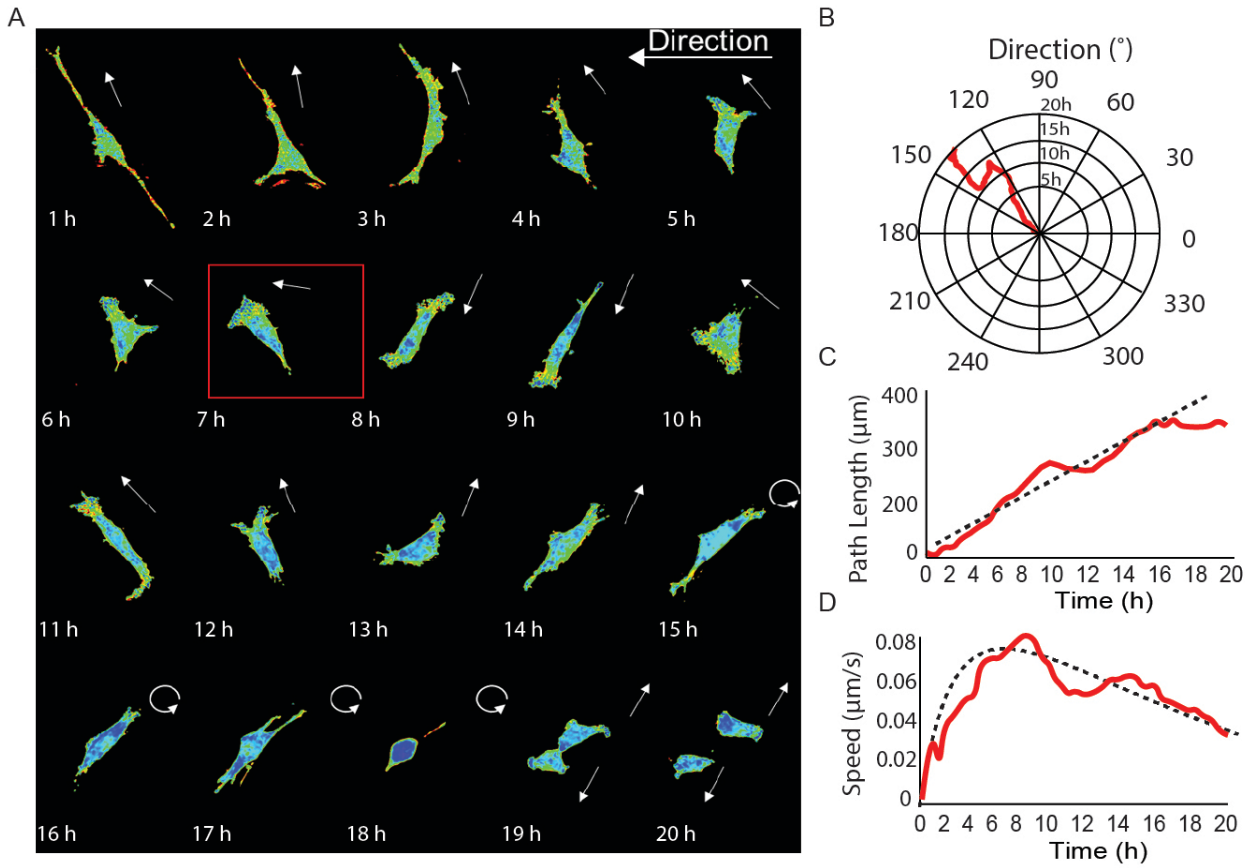

Fig. S2. Cell migration on fibronectin-coated mPADs. (**A**) Representative time-lapse images of a cell migrating over a 20 hour period. Arrows reflect instantaneous direction of migration. Representative metrics for a cell path showing persistent directional migration: (**B**) direction of migration, (**C**) path length, and (**D**) cell speed.

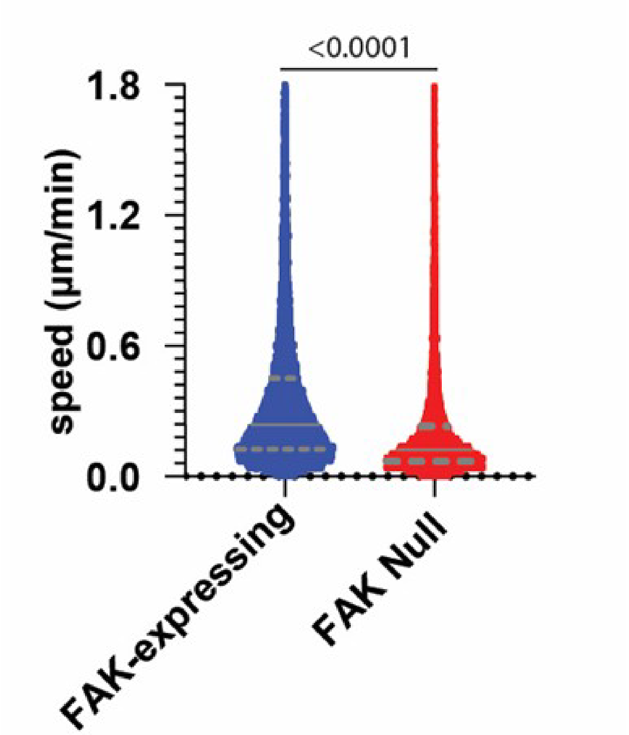

Fig. S3. Migration speed for FAK-null and FAK-expressing cells. Violin plots depicting cell migration speeds over a 20-hour period on stiff substrates (14 kPa) for FAK-expressing and FAK-null cells. The solid line within each violin represents the median speed, while dashed lines indicate the first and third quartiles. Statistical differences between groups were assessed using Mann-Whitney tests.

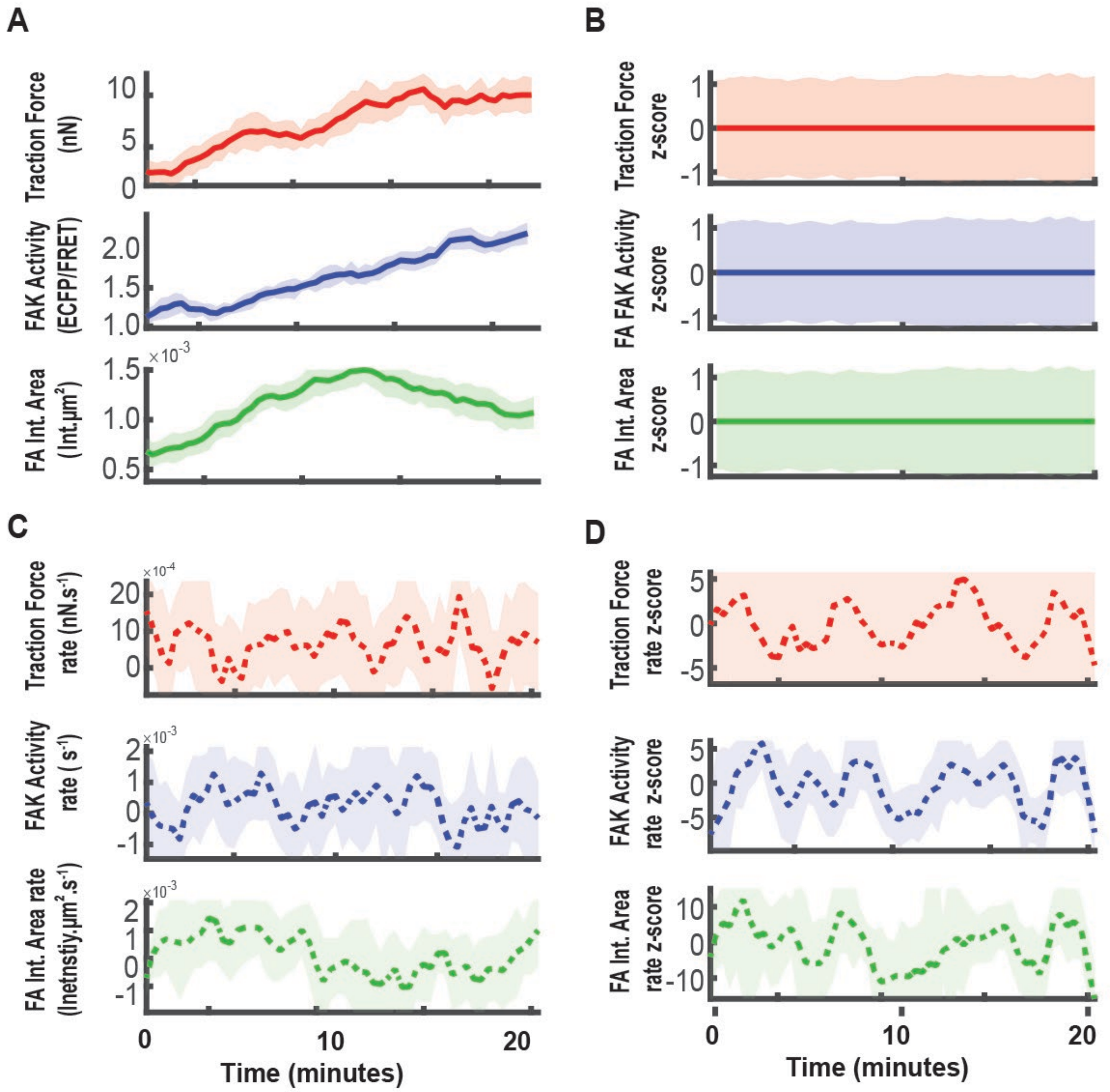

Fig. S4. Z-score normalization method for inter-FA metrics. (**A,B**) Original (non-normalized) temporal profile of each FA metric for measuring differences across same FA metric. (**C,D**) Z-score normalization centers the data for all FAs in a cell to 0 and sets a common scale (std. dev. = 1) to allow inter-F metric comparisions.

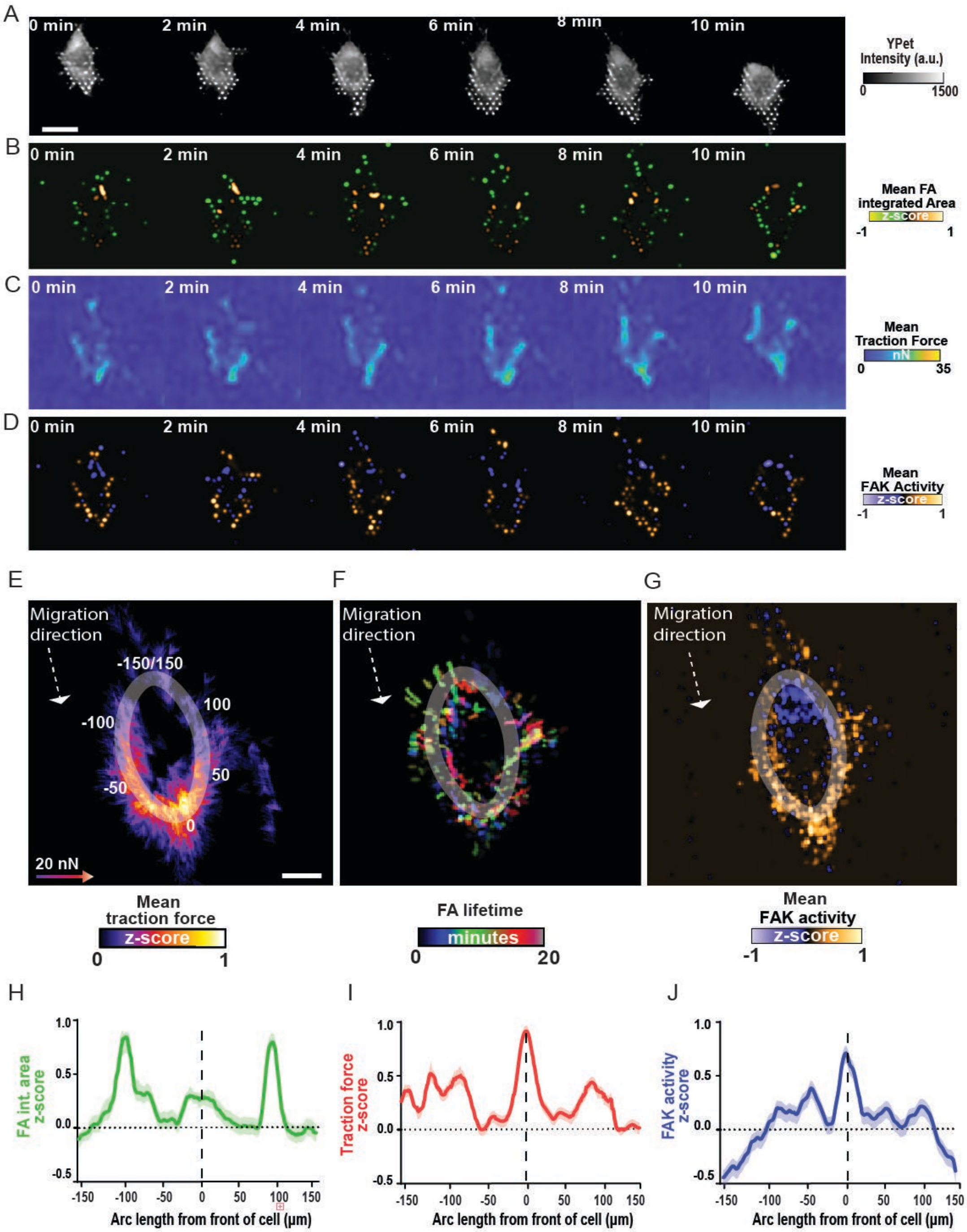

Fig. S5. Cell migration spatial patterns. (**A**) Time-lapse images showing the directional migration of a representative cell over a 10-minute period. Corresponding time-lapse images for (**B**) z-scores of FA integrated area, (**C**) traction force, and (**D**) z-scores of FAK activity. Time-averaged images showing spatial patterns of (**E**) mean traction force, (**F**) FA age, and (**G**) mean FAK activity. Quantitative analysis of spatial patterns for (**H**) FA integrated area, (**I**) traction force, and (**J**) FAK activity, illustrating the spatial coordination of mechanical and signaling events that facilitate directional movement. Data is presented as mean ± SD.

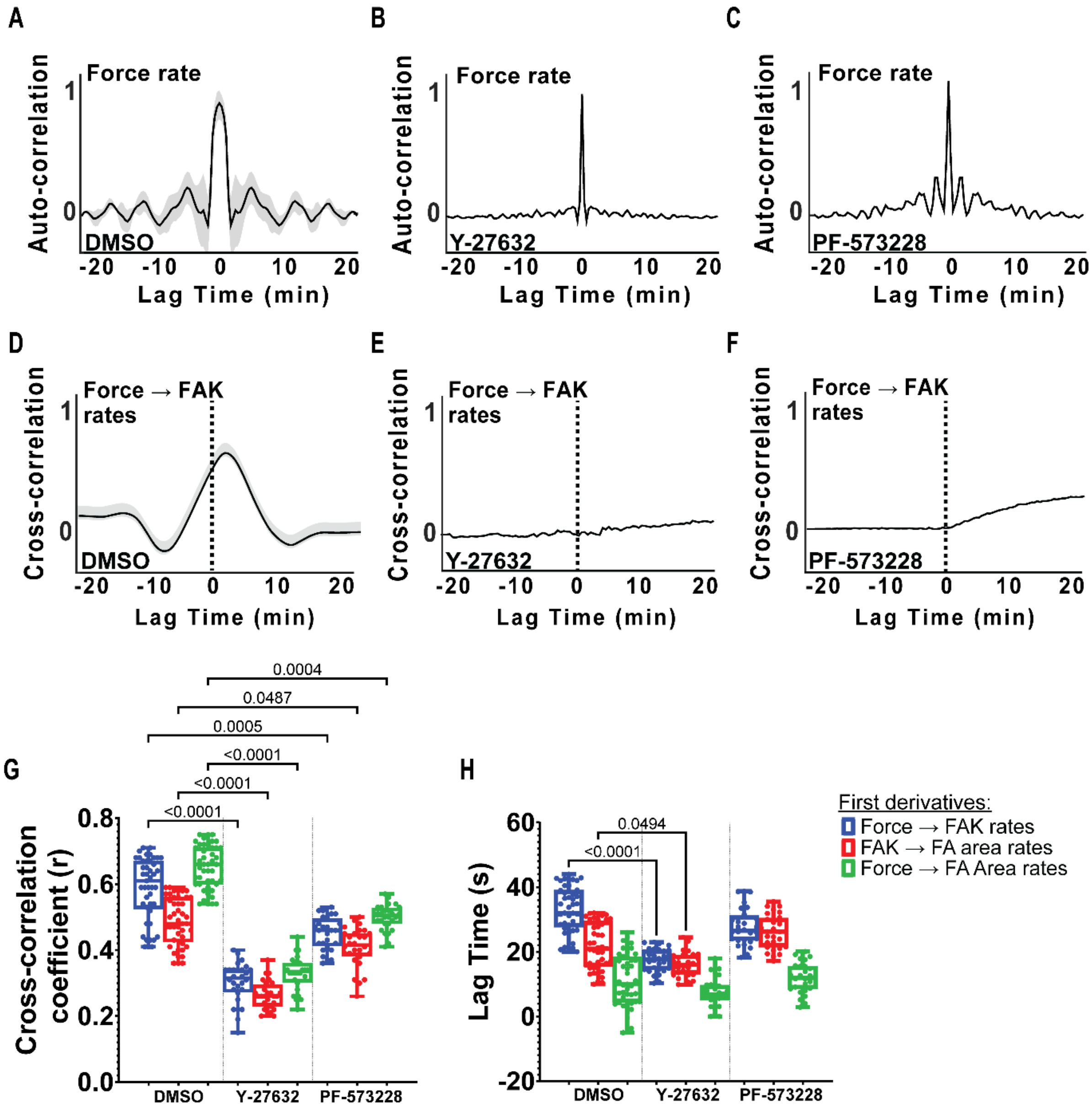

Fig. S6. **Temporal relationship among the rates of traction force, FAK activity, and FA integrated area.** Auto-correlation analyses for rates of traction force, FAK activity and, FA integrated area for (**A**) DMSO, (**B**) Y-27632 and (**C**) PF-573228. Oscillations with a periodicity of 4-5 minutes are only present in control cells (DMSO). Cross-correlation analyses for rates of traction force, FAK activity and, FA integrated area for (**D**) DMSO, (**E**) Y-27632 and (**F**) PF-573228. **(G)** Cross-correlation coefficients quantifying the strength of coupling between traction force and FAK (blue), FAK and FA area (red), and traction force and FA area (green) across treatment conditions. (**H**) Lag times extracted from cross-correlation peaks. Positive lag times indicate that the first signal leads the second (e.g., force → FAK). Data represent n=45 FAs (DMSO), n=28 FAs (Y-27632), and n=28 FAs (PF-573228) obtained from 9 cells across 4 independent experiments. Statistical analysis was performed with Kruskal-Wallis test, followed by Dunn's post-hoc multiple comparisons.

Captions for supplementary movies:

Movie S1. FAs in migrating cells.

Movie S2. Pillar deflections in migrating cells.

Movie S3. Traction force maps in migrating cells.

Movie S4. FAK activity in migrating cells.
